# Preoperative CT Assessment and Perioperative Decision-Making in Gallbladder Lesions: A Descriptive Pathway Analysis

**DOI:** 10.3390/diagnostics16213678

**Published:** 2026-11-06

**Authors:** Byeong Gwan Noh, Eun Ji Park, Young Mok Park, Myunghee Yoon, Hyung Il Seo, Myeong Hun Oh, Sunho Ok, Mingyu Kim, Suk Kim, Seung Baek Hong

**Affiliations:** 1 Department of Surgery, Biomedical Research Institute, Pusan National University Hospital, Busan 49241, Republic of Korea; sagerbk@naver.com (B.G.N.);; 2 Department of Surgery, School of Medicine, Pusan National University, Yangsan 50612, Republic of Korea; 3 Department of Anesthesia and Pain Medicine, Biomedical Research Institute, Pusan National University Hospital, Busan 49241, Republic of Korea; eangelj1@naver.com (E.J.P.);; 4 Department of Anesthesia and Pain Medicine, School of Medicine, Pusan National University, Yangsan 50612, Republic of Korea; 5 Department of Radiology, Biomedical Research Institute, Pusan National University Hospital, Busan 49241, Republic of Korea; 6 Department of Radiology, School of Medicine, Pusan National University, Yangsan 50612, Republic of Korea

**Keywords:** gallbladder cancer, computed tomography, diagnosis, uncertainty, cholecystectomy, clinical decision-making

## Abstract

**Background/Objectives:** Preoperative CT may not reliably distinguish gallbladder malignancy from inflammatory disease, complicating surgical planning. We examined transitions from CT impressions through operative plans, intraoperative reassessment, index procedures, and subsequent treatment. **Methods:** This retrospective descriptive study screened 651 consecutive patients who underwent gallbladder surgery by a single surgeon at a tertiary referral center from January 2023 to December 2025. The analytic cohort included 41 patients with indeterminate or suspicious CT impressions or malignancy on final pathology. CT categories were assigned from original radiology reports independently of operative intent and pathology. **Results:** CT impressions were benign in six patients, indeterminate in 11, and suspicious in 24; malignancy was confirmed in six, three, and 16, respectively. Among indeterminate cases, eight had simple cholecystectomy plans, two frozen-guided plans, and one a radical-possible plan; all three malignant cases had conditional oncologic plans. Frozen section was performed in 11 patients, identifying malignancy in nine. Of 19 extended index procedures, 18 were performed under plans allowing oncologic extension; one represented an unanticipated extension. Six patients with T1b or more advanced cancer initially underwent simple cholecystectomy; three subsequently underwent completion surgery. **Conclusions:** Patients with the same CT impression followed different operative and subsequent management pathways. These findings highlight the clinical importance of considering diagnostic uncertainty throughout the surgical course, from preoperative planning and intraoperative reassessment to subsequent treatment decisions informed by final pathology.

## 1. Introduction

Gallbladder cancer is an uncommon but aggressive malignancy with marked geographic variation and poor prognosis, particularly in advanced-stage disease [1,2]. In clinical practice, however, one of the main challenges is that benign inflammatory gallbladder disease may closely mimic malignancy on imaging, making reliable preoperative distinction difficult [3]. Preoperative computed tomography (CT) is therefore widely used in the evaluation of suspicious gallbladder lesions, although its diagnostic performance remains imperfect [4]. Recent reviews have also emphasized that accurate diagnosis and staging of gallbladder cancer remain challenging despite advances in imaging [5]. Because the surgical approach to suspicious gallbladder lesions may range from simple cholecystectomy to radical resection, this uncertainty has direct implications for operative planning.

Previous imaging studies have examined the differentiation of benign and malignant gallbladder lesions and the assessment of tumor extent [4,6,7,8]. Wall thickening, enhancement patterns, and signs of local extension may suggest malignancy [6,7], although imaging assessment of nodal metastasis remains limited [8]. Diagnostic performance estimates also depend on disease prevalence and cohort composition [9]. Alongside these diagnostic questions, the sequence from the reported CT impression to the documented operative plan and actual procedure warrants description.

Operative plans may include contingencies based on gross findings or frozen-section results. Frozen section can assist intraoperative decisions, but has practical limitations [10]. Previous surgical studies and recommendations have addressed the selection of operative extent according to disease stage and intraoperative assessment [11,12,13]. Malignancy identified after cholecystectomy introduces a further decision regarding completion surgery [14,15]. The index operation and subsequent management therefore need to be considered separately when describing the treatment course.

We examined these clinical transitions in a selected cohort with indeterminate or suspicious CT impressions or malignancy on final pathology. CT categories were based on the original clinical reports, independently of operative intent and pathological findings. We linked each CT category to the documented plan, intraoperative assessment, actual index procedure, final pathology, and subsequent management. Operative burden and postoperative outcomes were summarized descriptively.

## 2. Materials and Methods

### 2.1. Study Design and Cohort Assembly

This retrospective observational study was conducted at Pusan National University Hospital, a tertiary referral center. Electronic medical records were reviewed for patients who underwent surgery for gallbladder lesions between January 2023 and December 2025. The reviewed records included preoperative clinical assessment, original preoperative radiology reports, operative records, and final pathology reports.

During the study period, 651 consecutive patients underwent surgery for gallbladder lesions by a single surgeon at Pusan National University Hospital. All patients underwent preoperative CT, and the original radiology reports and final pathology were reviewed for eligibility. Patients were included if CT findings were indeterminate or suspicious for malignancy, or if malignancy was confirmed on final pathology. The 610 patients with benign CT impressions and nonmalignant final pathology were excluded, leaving 41 eligible patients. No additional patients were excluded because of unavailable imaging or insufficient records.

This single-surgeon setting reduced inter-operator variability in intraoperative decision-making and surgical escalation, although it may limit the generalizability of the findings. In particular, the observed pathway patterns may partly reflect surgeon-specific thresholds for intraoperative suspicion, frozen-section use, and escalation to radical resection.

### 2.2. CT Classification and Clinical Pathway Assessment

Preoperative CT impressions were classified as benign, indeterminate, or suspicious based on the final impression in the original clinical radiology report. Reports favoring benign or inflammatory disease without concern for malignancy were classified as benign. Reports describing equivocal findings or stating that malignancy could not be excluded were classified as indeterminate. Reports explicitly favoring gallbladder cancer or malignancy were classified as suspicious. Operative intent and final pathology were not used to assign CT categories. All indeterminate impressions were retained as a separate category.

Preoperative operative intent was classified from the documented surgical plan into three categories: a simple cholecystectomy plan, a frozen-guided plan, or a radical-possible plan. A simple cholecystectomy plan specified cholecystectomy without a documented oncologic extension. A frozen-guided plan indicated a conditional strategy in which frozen-section findings were expected to guide operative extent, although frozen-section examination could be omitted according to intraoperative findings. Operative extent could also be limited by patient-specific clinical considerations. A radical-possible plan explicitly included potential radical resection without requiring frozen-section confirmation, although frozen section could still be performed. When both approaches were considered, classification depended on whether frozen-section findings were intended as the primary guide to operative extent. These categories described preoperative intent, irrespective of the procedure ultimately performed. The operating surgeon explained the planned surgical procedure and relevant clinical findings to the patient and family during the preoperative surgical consent process, usually on the day before surgery. The possibility of changing the surgical approach or extent according to intraoperative findings was also discussed and documented. Operative plans were determined by the surgeon using the available imaging and clinical findings rather than through a routine formal multidisciplinary meeting.

Clinical pathways were reconstructed by linking CT impression, documented operative intent, intraoperative findings, frozen-section use and result, actual index procedure, final pathology, and subsequent management. These stages were recorded separately and summarized descriptively. No study-specific standardized malignancy scoring or reporting scheme was applied to the original clinical CT reports.

### 2.3. Clinical, Laboratory, and Preoperative Imaging Variables

Baseline variables included age, sex, body mass index, symptomatic presentation, acute cholecystitis, gallstone status, previous endoscopic retrograde cholangiopancreatography/endoscopic retrograde biliary drainage, and previous percutaneous transhepatic gallbladder drainage. Preoperative laboratory variables included carcinoembryonic antigen and carbohydrate antigen 19-9. These variables were summarized according to preoperative CT impression. All CT examinations were performed at our institution using a three-phase contrast-enhanced protocol comprising arterial, portal venous, and delayed phases. Images were reconstructed at a section thickness of 3 mm. Contrast volume was adjusted according to body weight, with an average of approximately 120 mL, and administered intravenously at 3.0–3.5 mL/s. Arterial, portal venous, and delayed-phase images were acquired approximately 40–45, 75–85, and 180–200 s after the start of contrast injection, respectively. CT examinations were interpreted during routine clinical care by two radiologists specializing in abdominal imaging. Classification was based on the final formal radiology report, without study-specific image reinterpretation. The interval from CT to the index operation was recorded. Magnetic resonance cholangiopancreatography (MRCP) was performed selectively according to the CT findings and clinical context. In patients with benign CT impressions, its principal purpose was to evaluate biliary anatomy and anatomical variants. Most patients with suspicious CT impressions underwent MRCP for further preoperative assessment, whereas its use in indeterminate cases was determined by the surgeon’s clinical judgment. MRCP was omitted in patients with contraindications to magnetic resonance imaging (MRI) or when comorbid conditions or limited tolerance precluded completion of the examination. Available MRCP findings were incorporated into operative planning; however, CT categories remained based solely on the final impressions in the original CT reports. Ultrasonography was not part of the routine preoperative protocol at our institution. Imaging-suspected T stage was categorized as not assigned, uncertain, T1–2, or ≥T3.

Morphologic CT variables included lesion pattern (wall thickening, polypoid lesion, or mass-replacing lesion), loss of the fat plane with the liver, adjacent liver change, regional lymph node presence, and pericholecystic fat stranding. These findings were summarized descriptively and were not used to override the CT category assigned from the final radiology report.

Data were extracted by one author and checked by the coauthors and corresponding author against the original radiology reports, operative records, pathology reports, and postoperative records. Discrepancies were resolved by rechecking the source records and reaching agreement through discussion. Undocumented findings were distinguished from explicitly negative findings and were not coded as negative solely because they were unmentioned. Findings that could not be determined from the records were retained as unknown; assessments not performed and items not applicable were recorded separately. The extracting author assigned CT categories from the final impressions in the formal clinical reports issued by the two abdominal radiologists; these assignments were checked by the coauthors and corresponding author against the source reports.

### 2.4. Intraoperative Decision Variables and Surgical Strategy

Intraoperative findings, frozen-section use and results, and actual procedures were extracted from operative and pathology records. Gross suspicion of malignancy was defined as the surgeon’s documented assessment before receipt of frozen-section results. Frozen-section findings and their influence on management were recorded separately from operative extent. For frozen-guided plans, the documented strategy allowed radical resection when malignancy was identified or the depth of invasion remained uncertain.

The index procedure was classified as simple cholecystectomy or an extended procedure, with hepatic resection, regional lymph node dissection, and other components recorded separately. Standard radical cholecystectomy comprised en bloc removal of the gallbladder with a wedge of the adjacent segment IVb/V liver bed, aiming for a 2-cm margin, and regional lymph node dissection. Procedures departing from this extent were described individually. Completion surgery after the index operation was recorded separately. Retrieval of regional nodes in a simple cholecystectomy specimen was not regarded as evidence of a formal lymph node dissection. Conversely, cholecystectomy with documented formal regional lymph node dissection was classified as an extended index procedure, even when hepatic resection was not performed.

The actual index procedure was compared with the documented preoperative plan. Execution of a frozen-guided or radical-possible contingency was distinguished from an unanticipated extension beyond the planned range. Intraoperative use or omission of frozen section was not used to retrospectively reclassify preoperative intent. Operative time, estimated blood loss, and Jackson-Pratt drain insertion were recorded as measures of operative burden. Blood loss documented as <50 mL was retained as a category without assigning a numerical value.

### 2.5. Pathologic and Postoperative Outcomes

Pathological outcomes included final diagnosis, T stage, regional lymph node assessment, and resection status. Tis, T1a, T1b, T2a, T2b, and T3 were reported separately. The total number of examined nodes and the number of metastatic nodes were recorded, distinguishing patients with negative examined nodes from those without nodal assessment. Nodal findings from the index operation and subsequent completion surgery were identified separately.

Resection status was classified as R0, R1, R2, or unknown, denoting no residual tumor, microscopic residual tumor, macroscopic residual tumor, or insufficient documentation to determine residual status, respectively. Final resection status incorporated completion surgery where performed. An operation lacking the usual components of radical resection was not classified as R2 without evidence of macroscopic residual disease.

Postoperative stay was defined as the interval from the index operation to discharge. Prolonged hospitalization was defined as a postoperative stay longer than seven days, with both measures derived from the same postoperative-stay variable. Postoperative complications and readmission within 30 days were recorded. Subsequent management included completion surgery and documented reasons for not undertaking further surgery. Outcomes were summarized descriptively according to the original CT category.

### 2.6. Statistical Analysis

Categorical variables were presented as counts and percentages, and continuous variables as medians and interquartile ranges. Quartiles were calculated using the type 7 method in R. Percentages were calculated within each CT category unless a subgroup denominator was specified. Given the small size and selected nature of the analytic cohort, all analyses were descriptive. No formal between-group hypothesis testing was performed, and *p*-values and diagnostic accuracy estimates are not reported. All descriptive analyses were conducted using R version 4.3.1 (R Foundation for Statistical Computing, Vienna, Austria). Findings classified as unknown, not assessed, or not applicable were retained as separate categories without imputation.

### 2.7. Pathway Visualization

An alluvial diagram linked the original CT impression, documented preoperative operative intent, actual index procedure, and final pathology. Flow widths represented patient counts, with numerical transition counts displayed on the ribbons. Intraoperative findings, frozen-section results, and subsequent management were detailed in Supplementary Table S1. Supplementary Figure S1 summarized the sequence from CT assessment through operative planning, intraoperative reassessment, the index procedure, final pathology, and subsequent management.

## 3. Results

Given the small size and selected nature of the analytic cohort, all group-wise summaries are presented descriptively, without formal inferential between-group testing.

### 3.1. Cohort Assembly and CT Classification

**Figure 1:**
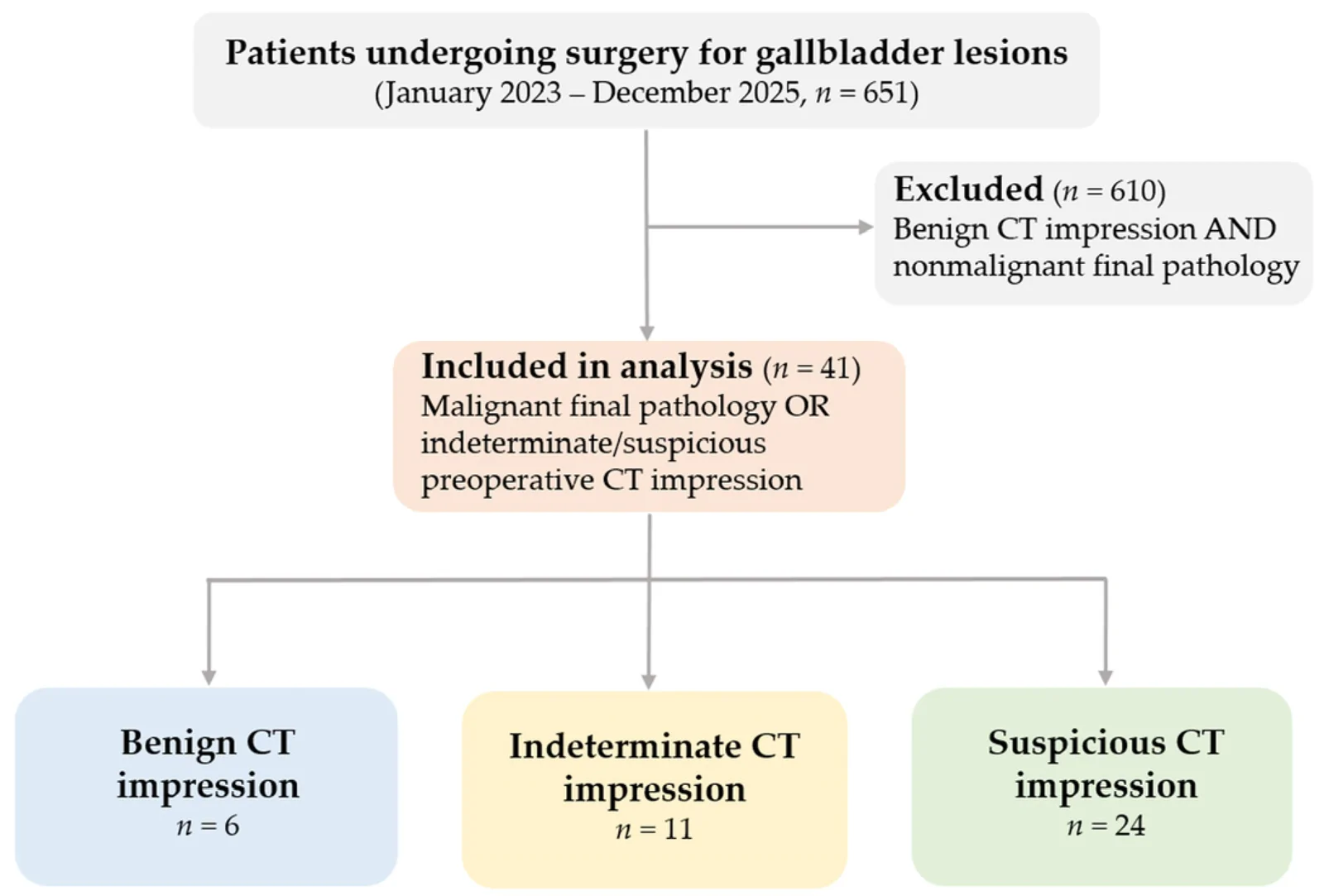
Cohort selection and classification by preoperative CT impression. All 651 consecutive gallbladder operations performed by a single surgeon at Pusan National University Hospital between January 2023 and December 2025 were screened. Patients with both a benign CT impression and nonmalignant final pathology were excluded (*n* = 610). The selected cohort comprised 41 patients with malignant final pathology or an indeterminate or suspicious CT impression. CT categories were based on the original final clinical radiology reports: benign (*n* = 6), indeterminate (*n* = 11), and suspicious (*n* = 24). No additional patients were excluded for unavailable CT or insufficient records. The selected cohort was not used to estimate CT diagnostic accuracy.

Among 651 patients who underwent surgery for gallbladder lesions during the study period, 610 had benign CT impressions and nonmalignant final pathology and were excluded. The remaining 41 patients comprised the analytic cohort: 6 had benign, 11 indeterminate, and 24 suspicious CT impressions. Malignancy was confirmed in all 6 patients with benign CT impressions, 3 of 11 with indeterminate impressions, and 16 of 24 with suspicious impressions (Figure 1).

### 3.2. Preoperative Clinical and Imaging Characteristics

Baseline clinical and preoperative imaging characteristics are summarized according to CT impression in Table 1. Wall thickening was the most common lesion pattern in all three categories, occurring in 5 of 6 benign, 9 of 11 indeterminate, and 13 of 24 suspicious cases. Mass-replacing lesions were confined to the suspicious category (8/24).

MRCP was performed in 27 of 41 patients: 1/6 with benign CT impressions, 9/11 with indeterminate impressions, and 17/24 with suspicious impressions. The median interval from CT to the index operation was 50 days (IQR, 24–65; range, 1–112).

**Figure 2:**
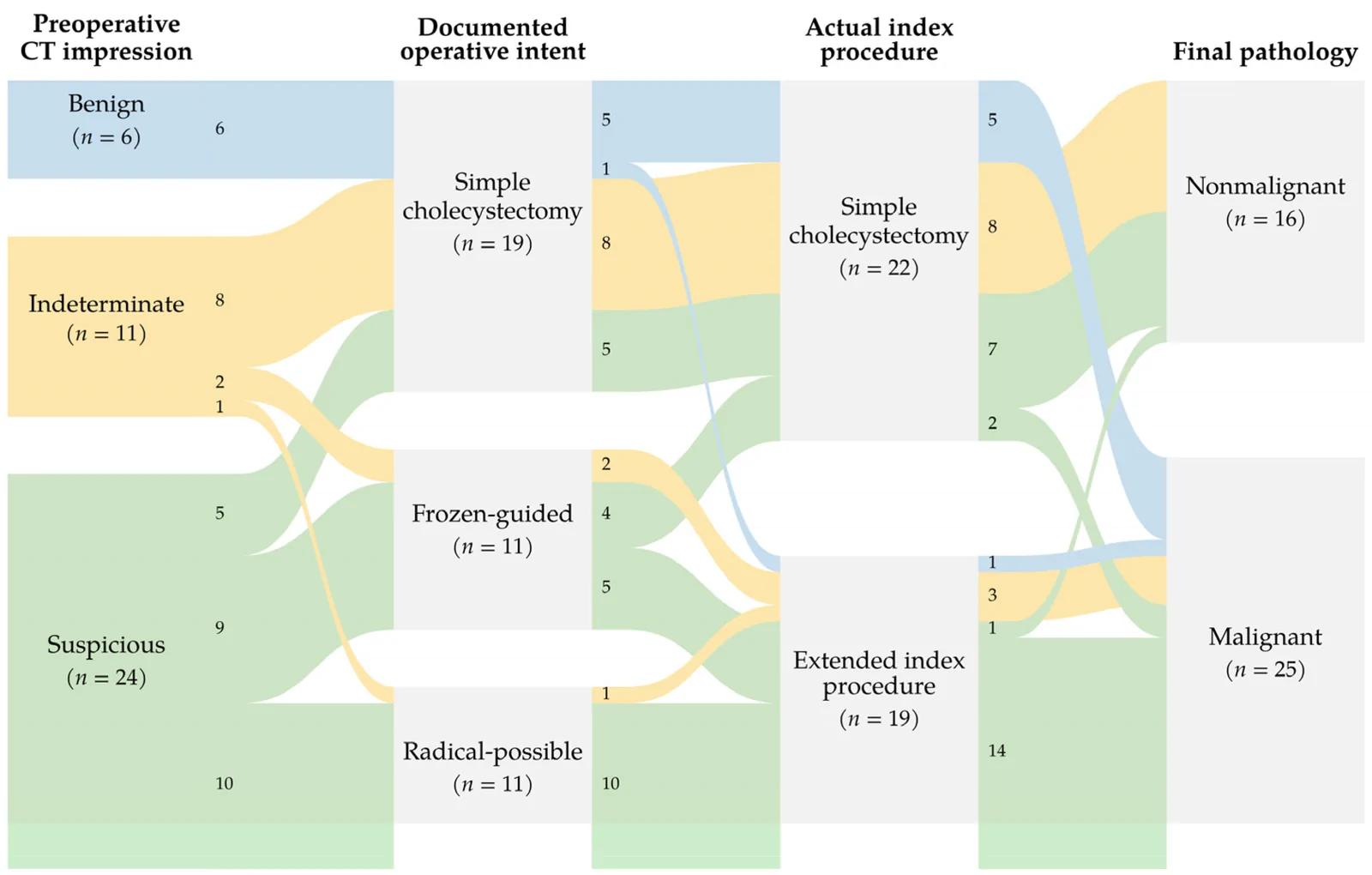
Observed transitions from CT impression to operative intent, index procedure, and final pathology. Ribbon colors indicate the original CT category; numbers on ribbons indicate patients within each colored transition. Preoperative intent comprised simple cholecystectomy (*n* = 19), a frozen-guided plan (*n* = 11), or a radical-possible plan (*n* = 11). Actual index procedures were simple cholecystectomy (*n* = 22) or an extended index procedure (*n* = 19). Extended procedures comprised cholecystectomy with liver wedge resection and regional lymph node dissection (LND; *n* = 16), radical cholecystectomy with combined left hepatectomy and LND (*n* = 1), cholecystectomy with LND without hepatic resection (*n* = 1), and cholecystectomy with LND and hepatic artery reconstruction without hepatic resection (*n* = 1). Arterial reconstruction followed an intraoperative injury. Frozen-guided denotes conditional preoperative intent, not a separate operative extent. Execution of a documented contingency is not classified as an unanticipated extension. Completion surgery is reported separately in Supplementary Table S1.

Simple cholecystectomy was planned in all 6 patients with benign CT impressions. Among the 11 patients with indeterminate impressions, 8 had a simple cholecystectomy plan, 2 a frozen-guided plan, and 1 a radical-possible plan. Among the 24 patients with suspicious impressions, the corresponding numbers were 5, 9, and 10. Transitions from CT impression through operative planning to surgery and final pathology are shown in Figure 2.

### 3.3. Intraoperative Reassessment and Actual Index Procedure

Intraoperative findings and frozen-section assessment are summarized in Table 2. Gross suspicion of malignancy was recorded in one of six patients with benign CT impressions, one of 11 with indeterminate impressions, and 15 of 24 with suspicious impressions. Frozen-section examination was performed in none of the patients with benign CT impressions, two of 11 with indeterminate impressions, and nine of 24 with suspicious impressions. Both indeterminate cases had malignant frozen-section results; among the suspicious cases, seven had malignant results and two had benign results.

The two patients with indeterminate CT impressions who underwent frozen-section examination had low intraoperative gross suspicion of malignancy, but examination was performed according to their documented frozen-guided plans. Invasion depth was not specified in either malignant frozen-section result, and both patients underwent cholecystectomy with liver wedge resection and regional lymph node dissection within the planned frozen-guided strategy. Final pathology showed T1a disease in one patient and Tis disease in the other.

At the index operation, simple cholecystectomy was performed in five patients with benign CT impressions, eight with indeterminate impressions, and nine with suspicious impressions. The corresponding numbers undergoing an extended index procedure were one, three, and 15, respectively. Overall, 22 patients underwent simple cholecystectomy and 19 underwent an extended index procedure.

When examined according to documented preoperative intent, 18 of 19 patients with a simple cholecystectomy plan underwent the planned procedure, whereas one underwent an extended index procedure. Among 11 patients with a frozen-guided plan, seven underwent an extended index procedure and four underwent simple cholecystectomy. Frozen section was performed in nine patients and omitted in two. In one patient, low gross suspicion led to simple cholecystectomy without frozen section, whereas in the other, gross suspicion prompted an extended procedure without frozen-section examination. Another patient underwent simple cholecystectomy despite a malignant frozen-section result because of poor baseline condition and high anesthetic risk. All 11 patients with a radical-possible plan underwent an extended index procedure. Overall, 18 of the 19 extended index procedures were performed under a documented frozen-guided (*n* = 7) or radical-possible (*n* = 11) plan. The remaining procedure represented an unanticipated oncologic extension from a simple cholecystectomy plan, prompted by intraoperative gross suspicion of malignancy and involving additional regional lymph node dissection without frozen-section examination.

Of the 19 extended index procedures, 16 comprised cholecystectomy with liver wedge resection and regional lymph node dissection. One patient underwent radical cholecystectomy with left hepatectomy and regional lymph node dissection for suspected left intrahepatic bile duct involvement. The patient with the unanticipated oncologic extension underwent emergency cholecystectomy with regional lymph node dissection but without hepatic resection for severe inflammatory disease. In the remaining patient, inflammatory changes complicated regional lymph node dissection, during which hepatic artery injury occurred and required unplanned reconstruction. Given the arterial reconstruction and absence of gross liver invasion, liver resection was omitted. This operation was classified as an extended index procedure because formal regional lymph node dissection was performed, whereas the arterial reconstruction represented an unplanned intraoperative event rather than an intended oncologic component.

Transitions between CT impression, documented preoperative intent, actual index procedure, and final pathology are shown in Figure 2, with patient-level details in Supplementary Table S1. Subsequent completion surgery was recorded separately.

### 3.4. Operative Burden and Postoperative Recovery

Operative variables are summarized according to CT impression in Table 2. Jackson-Pratt drains were placed in 2 of 6 patients with benign CT impressions, 3 of 11 with indeterminate impressions, and 19 of 24 with suspicious impressions.

Postoperative outcomes are summarized in Table 3. Median postoperative stay was 5.0 days (IQR, 2.8–5.8) in the benign CT group, 3.0 days (IQR, 1.0–5.0) in the indeterminate group, and 8.0 days (IQR, 5.8–9.0) in the suspicious group. Postoperative stays longer than seven days occurred in 1/6, 2/11, and 14/24 patients, respectively. Postoperative complications occurred in 0/6, 0/11, and 4/24 patients, and 30-day readmissions occurred in 1/6, 0/11, and 3/24 patients, respectively. These outcomes were described without formal between-group comparisons.

### 3.5. Pathologic Outcomes

Malignancy was confirmed in all 6 patients with benign CT impressions, 3 of 11 with indeterminate impressions, and 16 of 24 with suspicious impressions. The benign CT category included one T1a, one T1b, two T2a, and two T3 cancers. The indeterminate category included one Tis, one T1a, and one T2b cancer. Among patients with suspicious CT impressions, six had T2a, six T2b, and four T3 disease.

Resection status, assessed after completion surgery where performed, was R0 in 22 patients, R1 in two, and unknown in one. No patient was classified as R2 after review of the source records. Regional lymph nodes were examined in 24 of 25 patients with malignancy; seven had nodal metastases and 17 had negative examined nodes. One patient had no lymph node assessment. Lymph node yields are reported in Table 3.

Both R1 cases had T3 cancer with microscopic margin involvement. One underwent emergency cholecystectomy with regional lymph node dissection but without hepatic resection for severe inflammatory disease, and the other underwent cholecystectomy with liver wedge resection and regional lymph node dissection. Further surgery was not performed in the former patient because of advanced disease, and medical oncology assessment was pursued. Resection status was unknown in one patient who underwent emergency cholecystectomy for gallbladder perforation because the available records did not establish residual tumor status.

Six patients with T1b or more advanced cancer initially underwent simple cholecystectomy. Three subsequently underwent completion surgery comprising liver wedge resection and regional lymph node dissection. Completion surgery was not performed in the remaining three patients because of early recurrence in one and refusal of further surgery in two. In the latter two patients, the index procedure was limited to simple cholecystectomy because of poor baseline condition and high anesthetic risk. One underwent simple cholecystectomy despite a malignant frozen-section result under a frozen-guided plan, whereas the other underwent planned simple cholecystectomy without frozen-section examination. Final pathology showed T2a cancer in both patients. Completion surgery was subsequently planned but declined by both patients.

The 16 nonmalignant diagnoses comprised chronic cholecystitis with or without cholelithiasis or adenomyomatosis (*n* = 5), xanthogranulomatous cholecystitis (*n* = 4), chronic active cholecystitis (*n* = 2), low-grade biliary intraepithelial neoplasia (*n* = 2), pyloric gland adenoma (*n* = 2), and acute cholecystitis (*n* = 1). One case of low-grade biliary intraepithelial neoplasia also showed acute cholecystitis. The only patient with nonmalignant final pathology who underwent an extended index procedure had a suspicious CT impression and underwent cholecystectomy with liver wedge resection and regional lymph node dissection under a radical-possible plan, based on intraoperative gross suspicion without frozen-section confirmation. Final pathology revealed chronic active cholecystitis.

## 4. Discussion

### 4.1. Preoperative CT and Operative Planning

In this selected surgical cohort, patients with the same preoperative CT impression followed different operative courses, reflecting variation in documented operative plans, intraoperative assessment, and subsequent management. This was particularly evident among the 11 patients with indeterminate CT impressions: eight had a simple cholecystectomy plan, two had a frozen-guided plan, and one had a radical-possible plan. Thus, an indeterminate CT impression did not correspond to a uniform operative plan. All three patients with malignancy in this category had a documented conditional oncologic plan, whereas the eight patients with a simple cholecystectomy plan had nonmalignant final pathology. This observed correspondence between operative intent and final pathology should be interpreted descriptively, as operative planning incorporated information beyond the original CT report and the subgroup was too small to assess discriminatory performance.

Preoperative differentiation of benign inflammatory disease from gallbladder malignancy remains difficult on CT [4], particularly in lesions presenting as wall thickening [3,4]. Previous imaging studies have focused on differentiating benign from malignant lesions and assessing tumor extent using features such as enhancement patterns and signs of local extension [6,7]. CT-based radiomics has also been investigated to improve lesion characterization [16]. The present study addressed a different aspect of clinical care by linking the original CT impression to documented operative intent, intraoperative assessment, actual procedures, and subsequent management. Magnetic resonance imaging (MRI) can provide complementary tissue characterization and assessment of local extension, while MRCP helps delineate biliary involvement [7]. In this cohort, MRCP findings were considered in clinical planning where available. The CT-based analysis therefore describes pathways beginning with the original CT report, rather than decisions made using CT alone, and does not compare the diagnostic performance of CT and MRI.

### 4.2. Intraoperative Reassessment and Operative Extent

Only one of the 19 extended index procedures represented an unanticipated oncologic extension beyond the documented preoperative plan. The remaining 18 procedures were performed under documented frozen-guided or radical-possible plans that allowed oncologic extension, highlighting the distinction between surgery performed within the anticipated oncologic scope and an unanticipated extension beyond the preoperative plan. The unplanned hepatic artery reconstruction following injury during lymph node dissection was a separate intraoperative event rather than an intended oncologic component.

Frozen-section examination can assist operative decision-making in suspected gallbladder malignancy [10], and previous studies have described stepwise strategies incorporating imaging and intraoperative pathology [12]. In two patients with indeterminate CT impressions and low gross suspicion of malignancy, frozen section identified malignancy without specifying the depth of invasion. Both underwent liver wedge resection and regional lymph node dissection under their documented frozen-guided plans; final pathology showed T1a disease in one patient and Tis disease in the other. These cases illustrate the uncertainty that may remain regarding invasion depth and operative extent even after malignancy is identified intraoperatively. In another patient, simple cholecystectomy was performed despite a malignant frozen-section result because of poor baseline condition and high anesthetic risk, illustrating how patient-specific clinical considerations could limit the extent of surgery despite pathological confirmation of malignancy.

Gross assessment also contributed to operative decisions when frozen section was not performed. One patient with a suspicious CT impression underwent an extended procedure under a radical-possible plan on the basis of gross suspicion without frozen-section confirmation, but final pathology revealed chronic active cholecystitis. Conversely, the frequent use of simple cholecystectomy among patients with nonmalignant final pathology does not establish that intraoperative reassessment prevented unnecessary radical surgery, because most of these patients already had a simple cholecystectomy plan.

### 4.3. Index Surgery and Subsequent Management

Management after an incidental diagnosis of gallbladder cancer requires consideration of tumor stage and the need for completion resection, as addressed in previous studies, consensus recommendations, and treatment reviews [13,14,17]. A nationwide analysis found that re-resection remained underused despite its association with improved survival [18]. In this series, six patients with T1b or more advanced cancer initially underwent simple cholecystectomy. Three subsequently underwent completion surgery, whereas three did not because of early recurrence in one patient and refusal of further surgery in two. In the latter two patients, poor baseline condition and high anesthetic risk had limited the index procedure, with completion surgery planned after final pathological assessment. Their subsequent refusal shows why the index procedure alone does not fully describe the intended or completed treatment course. The patient with a benign CT impression who underwent unanticipated regional lymph node dissection during emergency cholecystectomy, as described above, had T3 cancer. Hepatic resection was not performed at the index operation, and no completion surgery was undertaken because of advanced disease.

Margin status also needs to be interpreted separately from operative extent. The three patients who underwent completion surgery had negative margins in the initial cholecystectomy specimens, but subsequently underwent liver resection and regional lymph node dissection. A negative margin after simple cholecystectomy therefore did not, by itself, establish that no further oncologic surgery was indicated. Two patients had R1 resections, and resection status remained unknown in one emergency case. Separating the index procedure, pathological findings, and subsequent management provides a more complete account of the treatment course than operative extent or resection status alone.

### 4.4. Operative Burden and Pathologic Severity

Patients with suspicious CT impressions had longer median operative times and postoperative stays, and more frequently underwent extended index procedures. All 16 patients with malignancy in this category had T2/T3 disease. The observed operative burden therefore needs to be interpreted in the context of the procedures performed and underlying disease severity, rather than attributed directly to the preoperative CT impression. Multicenter studies have documented variation in perioperative outcomes after gallbladder cancer surgery [19,20], and comparisons of hepatic resection techniques have also reported differences in blood loss and postoperative complications [21].

Postoperative complications occurred in 0/6 patients with benign CT impressions, 0/11 with indeterminate impressions, and 4/24 with suspicious impressions. The corresponding 30-day readmission counts were 1/6, 0/11, and 3/24. These small numbers do not support conclusions about equivalence or comparative safety. Rather, the operative and postoperative outcomes provide descriptive context for the different procedures undertaken within each CT category, while differences in surgical extent and pathological severity limit their interpretation as consequences of CT classification.

### 4.5. Limitations and Clinical Implications

This study has several limitations. First, it was a retrospective, single-center study with a small analytic cohort, and all operations were performed by one surgeon. Although this provided a relatively consistent setting for reconstructing perioperative decisions, the observed patterns may reflect individual judgment and local practice. Second, although all 651 surgical cases were screened, patients with both benign CT impressions and nonmalignant final pathology were excluded. The resulting cohort does not represent the full spectrum of gallbladder disease encountered in surgical practice, and the observed proportions of malignancy within CT categories cannot be interpreted as estimates of diagnostic accuracy or predictive value.

Third, CT impressions were classified from original clinical radiology reports without independent image reinterpretation, and interobserver agreement was not assessed. This approach preserved the information available during routine clinical care, but classification may vary with reporting practice. MRCP and other clinical findings also contributed to operative planning, so the independent influence of CT could not be isolated. We did not systematically assess discordance between CT and MRCP or determine which modality was given greater weight when their findings differed. Ultrasonography was not part of the routine preoperative protocol, and operative plans were not routinely established through formal multidisciplinary meetings, which may limit applicability to institutions with different assessment practices. Retrospective reconstruction was necessarily restricted to documented findings and decisions. Finally, the small sample size precluded meaningful adjustment for operative extent, pathological stage, and patient condition, limiting the interpretation of perioperative outcomes.

Despite these limitations, reconstructing patient-level transitions allowed planned oncologic contingencies to be distinguished from changes beyond the documented operative plan and captured treatment decisions extending beyond the index operation. These distinctions suggest that future evaluations of suspected gallbladder malignancy should consider not only imaging findings and operative extent but also the plans underlying surgical decisions and whether subsequent oncologic management was undertaken. Larger multicenter studies are needed to examine the generalizability and clinical consequences of these pathways.

## 5. Conclusions

In this selected surgical cohort, patients with the same preoperative CT impression followed different operative and subsequent management pathways. These pathways reflected documented operative plans, intraoperative reassessment, and patient-specific considerations, while final pathology informed decisions regarding further treatment. These findings highlight the importance of considering diagnostic uncertainty across the entire surgical course, from preoperative planning to subsequent oncologic management, rather than focusing solely on CT–pathology concordance or the index operation.

## Figures and Tables

**Table 1:** Baseline clinical, laboratory, and imaging characteristics according to preoperative CT impression. Data are presented as *n* (%) or median (IQR). Percentages use the CT-category denominator. CT-to-surgery interval refers to the index operation. Wall thickening, polypoid, and mass-replacing patterns describe the dominant morphology; other imaging features may coexist. Lymph node detection does not imply metastasis. All comparisons are descriptive. IQR, interquartile range; CT, computed tomography; MRCP, magnetic resonance cholangiopancreatography; BMI, body mass index; ERCP, endoscopic retrograde cholangiopancreatography; ERBD, endoscopic retrograde biliary drainage; PTGBD, percutaneous transhepatic gallbladder drainage; CEA, carcinoembryonic antigen; CA19-9, carbohydrate antigen 19-9.

Variable	Benign (*n* = 6)	Indeterminate (*n* = 11)	Suspicious (*n* = 24)
Demographics			
Age, years, median (IQR)	71.5 (65.0–78.0)	65.0 (53.5–73.5)	71.5 (65.5–80.0)
Male sex, *n* (%)	3 (50.0)	3 (27.3)	15 (62.5)
BMI, kg/m^2^, median (IQR)	22.36 (20.03–24.44)	23.91 (23.14–26.31)	22.98 (20.81–24.53)
Clinical presentation			
Symptomatic, *n* (%)	3 (50.0)	1 (9.1)	5 (20.8)
Acute cholecystitis, *n* (%)	3 (50.0)	1 (9.1)	6 (25.0)
Gallstone present, *n* (%)	4 (66.7)	4 (36.4)	8 (33.3)
Previous ERCP/ERBD, *n* (%)	1 (16.7)	0 (0.0)	3 (12.5)
Previous PTGBD, *n* (%)	1 (16.7)	0 (0.0)	2 (8.3)
Laboratory findings			
CEA, ng/mL, median (IQR)	1.87 (1.45–2.48)	2.00 (1.25–2.45)	2.40 (1.48–3.93)
CA19-9, U/mL, median (IQR)	7.85 (6.11–11.19)	7.94 (3.92–10.10)	9.63 (5.94–35.33)
Preoperative imaging			
MRCP performed, *n* (%)	1 (16.7)	9 (81.8)	17 (70.8)
CT-to-surgery interval, days, median (IQR)	66.0 (49.3–85.0)	50.0 (31.5–73.0)	39.5 (23.8–61.0)
Imaging-suspected T stage, *n* (%)			
Not assigned	6 (100.0)	5 (45.5)	0 (0.0)
Uncertain	0 (0.0)	6 (54.5)	13 (54.2)
T1–2	0 (0.0)	0 (0.0)	4 (16.7)
≥T3	0 (0.0)	0 (0.0)	7 (29.2)
Preoperative operative intent, *n* (%)			
Simple cholecystectomy	6 (100.0)	8 (72.7)	5 (20.8)
Frozen-guided plan	0 (0.0)	2 (18.2)	9 (37.5)
Radical-possible plan	0 (0.0)	1 (9.1)	10 (41.7)
Imaging morphology, *n* (%)			
Wall thickening	5 (83.3)	9 (81.8)	13 (54.2)
Polypoid lesion	1 (16.7)	2 (18.2)	3 (12.5)
Mass-replacing lesion	0 (0.0)	0 (0.0)	8 (33.3)
Blurred or lost fat plane with liver	1 (16.7)	4 (36.4)	15 (62.5)
Adjacent liver change	0 (0.0)	0 (0.0)	7 (29.2)
Regional lymph node present	2 (33.3)	0 (0.0)	12 (50.0)
Pericholecystic fat stranding	1 (16.7)	5 (45.5)	15 (62.5)

**Table 2:** Intraoperative findings, index procedures, and operative details according to preoperative CT impression. Data are presented as *n* (%) or median (IQR). Gross suspicion refers to recorded operative concern before frozen-section results. * Percentages are calculated among patients who underwent frozen-section examination. † Percentages use extended index procedures as the denominator (*n* = 1, 3, and 15). LND, regional lymph node dissection. Liver wedge denotes en bloc gallbladder-bed resection of segments IVb/V with a planned 2-cm margin. Procedures refer to the index operation. Two malignant frozen sections did not specify invasion depth. Extended index procedures comprise the four component categories shown; artery reconstruction followed an intraoperative arterial injury, and liver resection was omitted in that patient. Execution of a planned oncologic contingency was distinguished from unplanned hepatic artery reconstruction following intraoperative injury. Blood loss recorded as <50 mL was retained as a category without numerical substitution. All comparisons are descriptive. IQR, interquartile range.

Variable	Benign (*n* = 6)	Indeterminate (*n* = 11)	Suspicious (*n* = 24)
Intraoperative assessment			
Gross suspicion of malignancy, *n* (%)	1 (16.7)	1 (9.1)	15 (62.5)
Frozen section performed, *n* (%)	0 (0.0)	2 (18.2)	9 (37.5)
Frozen-section result, *n* (%) *			
Benign	—	0 (0.0)	2 (22.2)
Malignant	—	2 (100.0)	7 (77.8)
Indeterminate	—	0 (0.0)	0 (0.0)
Actual procedure at index surgery, *n* (%)			
Simple cholecystectomy	5 (83.3)	8 (72.7)	9 (37.5)
Extended index procedure	1 (16.7)	3 (27.3)	15 (62.5)
Components of extended index procedures, *n* (%) †			
Cholecystectomy + liver wedge + LND	0 (0.0)	3 (100.0)	13 (86.7)
Radical cholecystectomy + left hepatectomy + LND	0 (0.0)	0 (0.0)	1 (6.7)
Cholecystectomy + LND without hepatic resection	1 (100.0)	0 (0.0)	0 (0.0)
Cholecystectomy + LND + artery reconstruction	0 (0.0)	0 (0.0)	1 (6.7)
Findings accompanying extended procedures, *n* (%) †			
Malignant frozen section	0 (0.0)	2 (66.7)	6 (40.0)
Gross suspicion without frozen section	1 (100.0)	1 (33.3)	9 (60.0)
Operative details			
Operative time, min, median (IQR)	52.5 (40.0–80.0)	55.0 (35.0–122.5)	227.5 (148.8–291.3)
Drain insertion, *n* (%)	2 (33.3)	3 (27.3)	19 (79.2)
Estimated blood loss, *n* (%)			
<50 mL	4 (66.7)	9 (81.8)	6 (25.0)
≥50 mL	2 (33.3)	2 (18.2)	18 (75.0)

**Table 3:** Pathologic findings, subsequent surgery, and postoperative outcomes according to preoperative CT impression. Data are presented as n (%) or median (IQR). * T stage, nodal status, and resection status are reported among patients with malignancy (n = 6, 3, and 16, respectively). Lymph node yield is summarized among these patients with at least one examined node; nodal findings from completion surgery are included for three patients in the benign CT category. Resection status includes completion surgery and does not indicate the oncologic comprehensiveness of the procedure; these three patients had negative index margins and final R0 status. † The denominator is patients with ≥T1b cancer following simple cholecystectomy (n = 4, 0, and 2, respectively). Among these six patients, completion surgery was not performed because of early recurrence in one patient and patient refusal in two. Separately, one patient with T3 cancer underwent cholecystectomy with regional lymph node dissection at the index operation and did not undergo further surgery because of advanced disease. Postoperative outcomes refer to the index operation; stay was measured from surgery to discharge, and prolonged stay was defined as >7 days. Unknown denotes indeterminate residual status in one patient and is retained as a category. All comparisons are descriptive. IQR, interquartile range.

Variable	Benign (*n* = 6)	Indeterminate (*n* = 11)	Suspicious (*n* = 24)
Pathologic outcomes			
Malignancy confirmed, *n* (%)	6 (100.0)	3 (27.3)	16 (66.7)
Pathologic T stage, *n* (%) *			
Tis	0 (0.0)	1 (33.3)	0 (0.0)
T1a	1 (16.7)	1 (33.3)	0 (0.0)
T1b	1 (16.7)	0 (0.0)	0 (0.0)
T2a	2 (33.3)	0 (0.0)	6 (37.5)
T2b	0 (0.0)	1 (33.3)	6 (37.5)
T3	2 (33.3)	0 (0.0)	4 (25.0)
Lymph node assessment, *n* (%) *			
Assessed, negative	4 (66.7)	2 (66.7)	11 (68.8)
Assessed, positive	1 (16.7)	1 (33.3)	5 (31.3)
Not assessed	1 (16.7)	0 (0.0)	0 (0.0)
Lymph node yield, median (IQR) *	6.0 (3.0–10.0)	7.0 (6.0–9.5)	8.0 (6.0–10.0)
Resection status, *n* (%) *			
R0	4 (66.7)	3 (100.0)	15 (93.8)
R1	1 (16.7)	0 (0.0)	1 (6.3)
R2	0 (0.0)	0 (0.0)	0 (0.0)
Unknown	1 (16.7)	0 (0.0)	0 (0.0)
Subsequent surgery			
≥T1b cancer after simple cholecystectomy, *n*	4	0	2
Completion radical surgery, *n* (%) †	3 (75.0)	—	0 (0.0)
Postoperative outcomes			
Postoperative stay, days, median (IQR)	5.0 (2.8–5.8)	3.0 (1.0–5.0)	8.0 (5.8–9.0)
Postoperative stay >7 days, *n* (%)	1 (16.7)	2 (18.2)	14 (58.3)
Overall complications, *n* (%)	0 (0.0)	0 (0.0)	4 (16.7)
30-day readmission, *n* (%)	1 (16.7)	0 (0.0)	3 (12.5)

## Data Availability

The data presented in this study are available on request from the corresponding author. The data are not publicly available due to privacy and ethical restrictions.

## Supplementary Materials

The following supporting information can be downloaded at: https://www.mdpi.com/article/10.3390/diagnostics16213678/s1, Figure S1: Framework for reconstructing clinical decision pathways; Table S1: Patient-level operative pathways and subsequent management.
