# Supplementary material for: Preoperative CT Assessment and Perioperative Decision-Making in Gallbladder Lesions: A Descriptive Pathway Analysis
Source: Diagnostics (Basel). 2026 Nov 6;16(21):3678. doi: 10.3390/diagnostics16213678 (PMC13762200; doi:10.3390/diagnostics16213678)
Supplement: Supplementary file 1 [file diagnostics-16-03678-s001.zip › FigureS1.pdf]

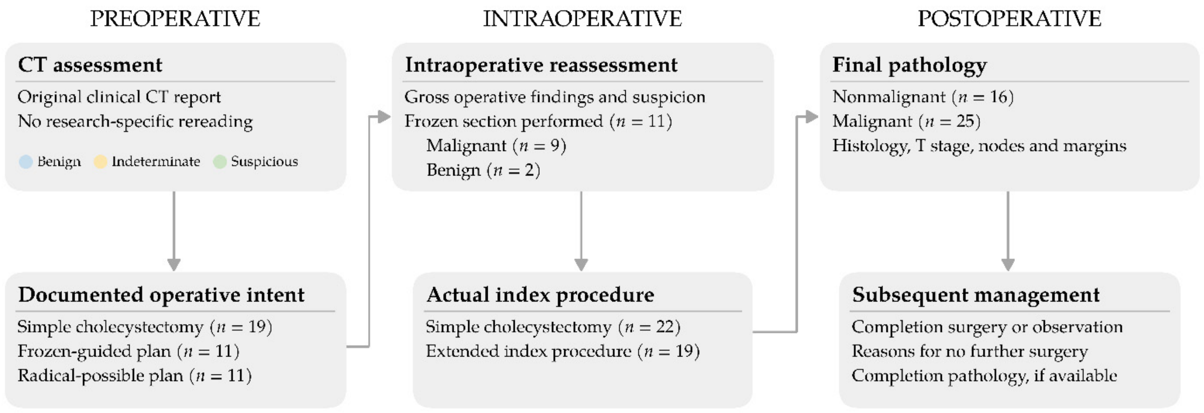

Figure S1. Framework for reconstructing clinical decision pathways. The framework separates original CT assessment, documented preoperative intent, intraoperative reassessment, actual index procedure, pathology, and subsequent management. Frozen-section examination and its result are recorded independently of operative extent. Extended index procedure has the same definition as in Figure 2 and Table 2. Index pathology and findings from completion surgery are distinguished in the patient-level table. Operative and postoperative outcomes describe the index operation. Arrows indicate clinical sequence, not causal effects or comparative effectiveness. CT, computed tomography.
