# Supplementary material for: Preoperative CT Assessment and Perioperative Decision-Making in Gallbladder Lesions: A Descriptive Pathway Analysis
Source: Diagnostics (Basel). 2026 Nov 6;16(21):3678. doi: 10.3390/diagnostics16213678 (PMC13762200; doi:10.3390/diagnostics16213678)
Supplement: Supplementary file 1 [file diagnostics-16-03678-s001.zip › Table S1.pdf]

Supplementary Table S1. Patient-level operative pathways and subsequent management.

A. Preoperative assessment and index surgery

Case	CT impression	Preoperative intent	Recorded gross suspicion	Frozen section	Index procedure	Decision and operative context
C01	Benign	Simple cholecystectomy	No	Not performed	Simple cholecystectomy	Planned simple procedure
C02	Benign	Simple cholecystectomy	No	Not performed	Simple cholecystectomy	Planned simple procedure
C03	Benign	Simple cholecystectomy	No	Not performed	Simple cholecystectomy	Emergency surgery for perforation and severe inflammation
C04	Benign	Simple cholecystectomy	Yes	Not performed	Cholecystectomy + LND; no hepatic resection	Emergency surgery for severe inflammatory disease; intraoperative gross suspicion of malignancy prompted additional regional LND
C05	Indeterminate	Frozen-guided	No	Malignant; invasion depth not specified	Cholecystectomy + liver wedge + LND	Planned conditional radical resection after malignant frozen; invasion depth not specified
C06	Benign	Simple cholecystectomy	No	Not performed	Simple cholecystectomy	Planned simple procedure
C07	Indeterminate	Frozen-guided	No	Malignant; invasion depth not specified	Cholecystectomy + liver wedge + LND	Planned conditional radical resection after malignant frozen; invasion depth not specified
C08	Benign	Simple cholecystectomy	No	Not performed	Simple cholecystectomy	Planned simple procedure
C09	Indeterminate	Simple cholecystectomy	No	Not performed	Simple cholecystectomy	Planned simple procedure
C10	Indeterminate	Simple cholecystectomy	No	Not performed	Simple cholecystectomy	Planned simple procedure
C11	Indeterminate	Simple cholecystectomy	No	Not performed	Simple cholecystectomy	Planned simple procedure
C12	Suspicious	Frozen-guided	No	Benign	Simple cholecystectomy	Simple procedure after benign frozen
C13	Indeterminate	Simple cholecystectomy	No	Not performed	Simple cholecystectomy	Planned simple procedure
C14	Suspicious	Simple cholecystectomy	No	Not performed	Simple cholecystectomy	Planned simple procedure

Supplementary Table S1 continued

A. Preoperative assessment and index surgery

Case	CT impression	Preoperative intent	Recorded gross suspicion	Frozen section	Index procedure	Decision and operative context
C15	Suspicious	Simple cholecystectomy	No	Not performed	Simple cholecystectomy	Planned simple procedure
C16	Indeterminate	Simple cholecystectomy	No	Not performed	Simple cholecystectomy	Planned simple procedure
C17	Suspicious	Frozen-guided	No	Benign	Simple cholecystectomy	Simple procedure after benign frozen
C18	Suspicious	Simple cholecystectomy	No	Not performed	Simple cholecystectomy	Planned simple procedure
C19	Indeterminate	Simple cholecystectomy	No	Not performed	Simple cholecystectomy	Planned simple procedure
C20	Suspicious	Simple cholecystectomy	No	Not performed	Simple cholecystectomy	Planned simple procedure
C21	Suspicious	Frozen-guided	No	Not performed	Simple cholecystectomy	Low gross suspicion; frozen omitted; simple procedure retained
C22	Suspicious	Radical-possible	Yes	Not performed	Cholecystectomy + liver wedge + LND	Radical-possible plan executed on gross suspicion, without frozen
C23	Indeterminate	Simple cholecystectomy	No	Not performed	Simple cholecystectomy	Planned simple procedure
C24	Indeterminate	Simple cholecystectomy	No	Not performed	Simple cholecystectomy	Planned simple procedure
C25	Suspicious	Frozen-guided	Yes	Malignant	Cholecystectomy + liver wedge + LND	Planned conditional radical resection after malignant frozen
C26	Suspicious	Radical-possible	Yes	Malignant	Cholecystectomy + liver wedge + LND	Radical-possible plan executed; malignant frozen
C27	Suspicious	Radical-possible	Yes	Malignant	Cholecystectomy + liver wedge + LND	Radical-possible plan executed; malignant frozen
C28	Suspicious	Frozen-guided	Yes	Malignant	Simple cholecystectomy	Limited index procedure because of poor baseline condition and high anesthetic risk

Supplementary Table S1 continued

A. Preoperative assessment and index surgery

Case	CT impression	Preoperative intent	Recorded gross suspicion	Frozen section	Index procedure	Decision and operative context
C29	Suspicious	Simple cholecystectomy	No	Not performed	Simple cholecystectomy	Limited index procedure because of poor baseline condition and high anesthetic risk
C30	Suspicious	Frozen-guided	Yes	Malignant	Cholecystectomy + liver wedge + LND	Planned conditional radical resection after malignant frozen
C31	Suspicious	Frozen-guided	No	Malignant	Cholecystectomy + liver wedge + LND	Planned conditional radical resection after malignant frozen
C32	Suspicious	Frozen-guided	Yes	Malignant	Cholecystectomy + liver wedge + LND	Planned conditional radical resection after malignant frozen
C33	Suspicious	Radical-possible	Yes	Not performed	Cholecystectomy + liver wedge + LND	Radical-possible plan executed; gross suspicion
C34	Suspicious	Radical-possible	Yes	Not performed	Cholecystectomy + LND + hepatic artery reconstruction; no liver resection	Planned oncologic procedure; unplanned artery repair after injury during LND; liver resection omitted
C35	Suspicious	Frozen-guided	Yes	Not performed	Cholecystectomy + liver wedge + LND	Gross suspicion prompted radical resection without frozen
C36	Suspicious	Radical-possible	Yes	Not performed	Cholecystectomy + liver wedge + LND	Radical-possible plan executed; gross suspicion
C37	Suspicious	Radical-possible	Yes	Not performed	Cholecystectomy + liver wedge + LND	Radical-possible plan executed; gross suspicion
C38	Indeterminate	Radical-possible	Yes	Not performed	Cholecystectomy + liver wedge + LND	Radical-possible plan executed; gross suspicion
C39	Suspicious	Radical-possible	Yes	Not performed	Radical cholecystectomy + left hepatectomy + LND	Radical-possible plan executed; left hepatectomy for suspected left intrahepatic bile duct involvement
C40	Suspicious	Radical-possible	Yes	Not performed	Cholecystectomy + liver wedge + LND	Radical-possible plan executed; gross suspicion
C41	Suspicious	Radical-possible	Yes	Not performed	Cholecystectomy + liver wedge + LND	Radical-possible plan executed; gross suspicion

CT, computed tomography; LND, lymph node dissection. Frozen-guided denotes a preoperative conditional strategy and does not imply that frozen examination was performed in every case. Gross suspicion was assessed before frozen results; No denotes low suspicion, not an undocumented finding.

Supplementary Table S1 continued
B. Pathology and subsequent management

Case	Final histology	T category	Positive/examined nodes	Resection status	Subsequent management
C01	Gallbladder cancer	T2a	0/10 (completion)	R0	Completion radical surgery with liver wedge resection and LND; nodal findings shown are from completion surgery
C02	Gallbladder cancer	T2a	0/6 (completion)	R0	Completion radical surgery with liver wedge resection and LND; nodal findings shown are from completion surgery
C03	Gallbladder cancer	T3	Not assessed	Unknown	No completion surgery because of early recurrence
C04	Gallbladder cancer	T3	5/19	R1	No completion surgery; advanced disease referred for medical oncology assessment
C05	Gallbladder cancer	T1a	0/5	R0	Index procedure detailed in panel A
C06	Gallbladder cancer	T1b	0/3 (completion)	R0	Completion radical surgery with liver wedge resection and LND; nodal findings shown are from completion surgery
C07	Gallbladder cancer	Tis	0/7	R0	Index procedure detailed in panel A
C08	Gallbladder cancer	T1a	0/1	R0	Observation without additional surgery for final T1a disease
C09	Chronic cholecystitis with cholelithiasis	Not applicable	Not assessed	Not applicable	Not applicable
C10	Low-grade biliary intraepithelial neoplasia	Not applicable	Not assessed	Not applicable	Not applicable
C11	Chronic cholecystitis	Not applicable	Not assessed	Not applicable	Not applicable
C12	Low-grade biliary intraepithelial neoplasia + Acute cholecystitis	Not applicable	Not assessed	Not applicable	Not applicable
C13	Chronic cholecystitis	Not applicable	Not assessed	Not applicable	Not applicable
C14	Xanthogranulomatous cholecystitis	Not applicable	Not assessed	Not applicable	Not applicable

Supplementary Table S1 continued

B. Pathology and subsequent management

Case	Final histology	T category	Positive/examined nodes	Resection status	Subsequent management
C15	Chronic active cholecystitis	Not applicable	Not assessed	Not applicable	Not applicable
C16	Chronic cholecystitis + adenomyomatosis	Not applicable	Not assessed	Not applicable	Not applicable
C17	Xanthogranulomatous cholecystitis	Not applicable	Not assessed	Not applicable	Not applicable
C18	Acute cholecystitis	Not applicable	Not assessed	Not applicable	Not applicable
C19	Xanthogranulomatous cholecystitis	Not applicable	0/1	Not applicable	Not applicable
C20	Pyloric gland adenoma	Not applicable	Not assessed	Not applicable	Not applicable
C21	Pyloric gland adenoma	Not applicable	Not assessed	Not applicable	Not applicable
C22	Chronic active cholecystitis	Not applicable	0/15	Not applicable	Not applicable
C23	Xanthogranulomatous cholecystitis	Not applicable	Not assessed	Not applicable	Not applicable
C24	Chronic cholecystitis + adenomyomatosis	Not applicable	Not assessed	Not applicable	Not applicable
C25	Gallbladder cancer	T2b	0/6	R0	Index procedure detailed in panel A
C26	Gallbladder cancer	T2b	1/10	R0	Index procedure detailed in panel A
C27	Gallbladder cancer	T2a	2/8	R0	Index procedure detailed in panel A
C28	Gallbladder cancer	T2a	0/2	R0	Completion surgery planned after final pathology; not performed because the patient declined

Supplementary Table S1 continued

B. Pathology and subsequent management

Case	Final histology	T category	Positive/examined nodes	Resection status	Subsequent management
C29	Gallbladder cancer	T2a	0/2	R0	Completion surgery planned after final pathology; not performed because the patient declined
C30	Gallbladder cancer	T2a	0/6	R0	Index procedure detailed in panel A
C31	Gallbladder cancer	T3	0/8	R1	Index procedure detailed in panel A
C32	Gallbladder cancer	T2b	2/8	R0	Index procedure detailed in panel A
C33	Gallbladder cancer	T3	2/20	R0	Index procedure detailed in panel A
C34	Gallbladder cancer	T2b	0/7	R0	Index procedure detailed in panel A
C35	Gallbladder cancer	T2a	0/9	R0	Index procedure detailed in panel A
C36	Gallbladder cancer	T3	0/14	R0	Index procedure detailed in panel A
C37	Gallbladder cancer	T2a	0/6	R0	Index procedure detailed in panel A
C38	Gallbladder cancer	T2b	6/12	R0	Index procedure detailed in panel A
C39	Gallbladder cancer	T3	0/11	R0	Index procedure detailed in panel A
C40	Gallbladder cancer	T2b	0/6	R0	Index procedure detailed in panel A
C41	Gallbladder cancer	T2b	6/10	R0	Index procedure detailed in panel A

Unless otherwise indicated, lymph-node findings were obtained at the index operation. For C01, C02, and C06, node counts are from completion surgery; index margins were negative and final status after completion was R0. Liver wedge denotes en bloc gallbladder-bed resection of segments IVb/V with a planned 2-cm margin. Node recovery does not by itself indicate formal LND. Resection status includes completion surgery and is not applicable to nonmalignant lesions; R0 does not establish oncologic comprehensiveness. Extended index procedures comprise liver wedge + LND (16), combined left hepatectomy + LND (1), cholecystectomy + LND without hepatic resection (1), and LND + artery reconstruction without liver resection (1). C03 has unknown residual status. C04 and C31 have positive pathologic margins (R1).
